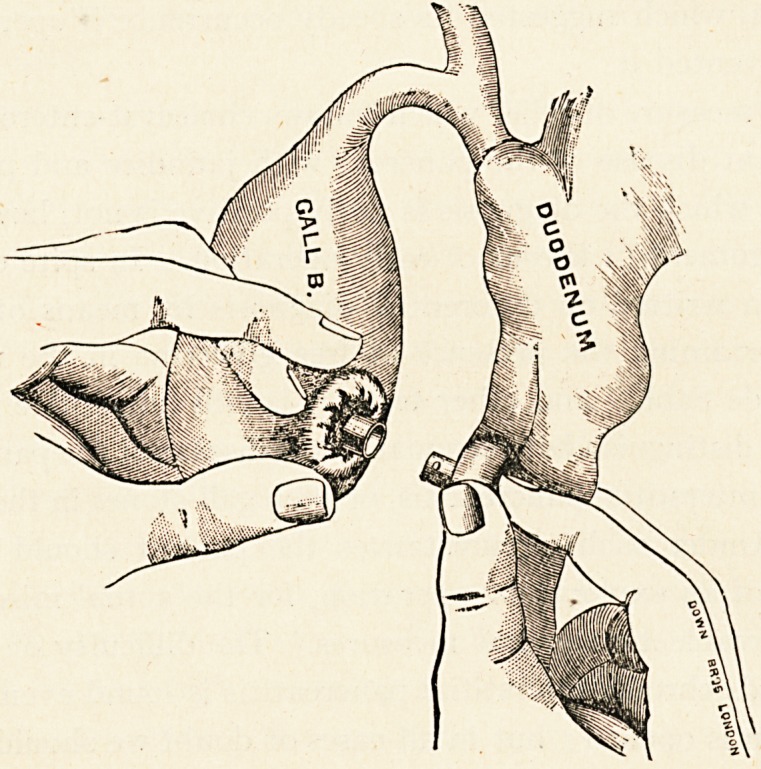# Operative Interference in Carcinoma of the Pancreas

**Published:** 1908-03

**Authors:** James Swain

**Affiliations:** Professor of Surgery in University College, Bristol; Surgeon to the Bristol Royal Infirmary


### OPERATIVE INTERFERENCE IN CARCINOMA OF THE PANCREAS.

BY

JAMES SWAIN, M.S. M.D. Lond., F.R.C.S.,

*Professor of Surgery in University College, Bristol;*
*Surgeon to the Bristol Royal Infirmary.*

---

PRIMARY carcinoma of the body or tail of the pancreas is capable of removal by a radical operation in the early stages; but inasmuch as jaundice is not present when the disease occurs in these situations, the diagnosis of the case at a sufficiently early period to justify such a procedure is very rare. When a tumour is palpable it is too late to consider the question of removal; and even under favourable circumstances the operative difficulties are so great, and the accompanying mortality so high, that we may well hesitate before adopting a method of treatment which is so uncertain in result.

Probably not 4 per cent. of cases of carcinoma of the pancreas are sufficiently isolated to render an attempt at radical cure justifiable, even if they could be discovered at an incipient stage of the disease.

Most cases of carcinoma of the pancreas occur in the head of the organ; and in this situation the presence of jaundice with distended gall-bladder, the rapid wasting and other symptoms, render the diagnosis more easy, but a radical operation is practically impossible.

The surgery of malignant disease of the pancreas is therefore mainly concerned with the performance of operations intended only to palliate certain symptoms, and, indirectly, to prolong life in cases which do not admit of the extirpation of the mischief. I propose, therefore, to discuss only this aspect of the subject.

Jaundice, as a result of pressure of the enlarged head of the pancreas upon the common bile-duct, is perhaps the most patent symptom. The cholæmia accompanying it is associated with depression and loss of appetite, and with the deposit of bile-pigment (bilirubin) in the skin, which is attended by considerable itching. This at times is intolerable, it is frequently worse at night, and the patient suffers from want of sleep. These conditions are productive of much misery, though fortunately some do not suffer materially from pruritus and the other phenomena of cholæmia; but when they obtain in a marked degree, the patient may well ask for relief from suffering, even though he knows himself to be afflicted by a mortal disease.

The evil effects of the damming back of the bile by the pressure of the infiltrated head of the pancreas is best obviated by sewing the gall-bladder on to some part of the intestine, and so forming a new route by which the bile can enter the alimentary canal. There are some conditions in which it might be preferable to attach the dilated common duct to the intestine (choledocho-enterostomy) instead, but such cases are so rare that they need not be further considered.

After operation the jaundice gradually disappears, and with that the general well-being of the patient improves. The pruritus ceases, the more natural colour prevents annoyance from the stare of the ignorant or curious observer, and the patient lives in comparative comfort until the final stage of the disease sets in. These points are exemplified in the following three typical cases, which may be referred to before considering other questions of importance on this subject.

**Case 1.**—E.H., female, aged 69 years, was admitted to the Bristol Royal Infirmary on January 17th, 1906. Eight weeks before admission she began to have diarrhœa, which continued in spite of treatment. A few days later she noticed that the urine was darker in colour than usual, and soon after this the skin became jaundiced, and gradually became deeper in colour. This was accompanied by an intolerable itching, but there was no pain. Vomiting occurred two or three times a day. Wasting set in rapidly, and she was so thin that the liver and gall-bladder could be plainly seen moving up and down with respiration. The liver was smooth, and extended nearly two inches below the costal margin; the gall-bladder was distinctly enlarged. There was no tenderness on palpation, and no tumour could be felt. The motions were copious, unformed, and of a pale greyish colour. The urine was acid, sp. gr. 1018, contained a good deal of albumin but no sugar, and was of a greenish-black colour from the presence of large quantities of bile. Examined for free glycerine by Cammidge's method, "A" was positive and "B" negative, thus suggesting that the disease was more likely of an inflammatory than a malignant nature. Nothing abnormal was felt on rectal examination. The patient was extremely weak, though there was a fair pulse of 72 per minute, and she was suffering from great depression. Her chief complaint was of the intense itching of the skin, which kept her awake at night and produced a condition of much misery.

On January 19th a vertical incision was made over the gall-bladder, which was enlarged and distended with bile. The common bile-duct was enormously dilated, and the head of the pancreas was enlarged, hard, and nodular. After emptying the gall-bladder by aspiration, it was attached to the duodenum (second part) by means of the Murphy button, and the abdomen was then closed.

Recovery from operation was uneventful, except for a slight temporary rise of temperature at night. No more vomiting occurred, diarrhœa ceased, bile began to appear in the motions six days after operation, and the colour of the skin gradually improved. The Murphy button was passed on the tenth day. The itching of the skin was very troublesome for the first few days after operation, then gradually became less, and disappeared entirely about three weeks after operation. The general condition greatly improved, appetite returned, and she was discharged on February 27th, 1907. At this time the liver dulness was still increased in area, but the gall-bladder could not be felt ; the colour of the skin had been steadily improving, but slight jaundice was present.

In answer to an inquiry, Dr. Spettigue informed me that digestive troubles began towards the end of October, but she was able to walk some distance till November. Shortly after this she " became more and more troubled with flatulence, and wasted more rapidly." There was some impaction of fæces on December 4th, and she died on December 8th, 1906. " There was no jaundice at any time, but the typical ashen look of malignancy marked towards the last."

**Case 2.**—Mrs. W., aged 71 years, had complained of not feeling well for about a year, and had occasional vomiting. On January 27th, 1907, there was a temperature of 101°—102° for about a week, accompanied by vomiting, slight jaundice and constipation. A similar attack occurred on February 5th, but this time the jaundice had become very marked, and continued so. The urine was deeply bile-stained, and the motions " white." The temperature varied between 98.4° and 101°. There was slight loss of flesh, great muscular weakness, and intense irritation of the skin. There had been no pain throughout.

I first saw the patient on February 25th. She was well nourished, and the pulse of good tone. There was mental depression, and the irritation of the skin was the cause of much discomfort. The liver was enlarged to halfway between the umbilicus and costal margin, its surface was smooth ; the gall-bladder was distended. Nothing abnormal was felt per rectum. She steadily became weaker, and the appetite began to fail.

On March 6th an incision was made in the right semi-lunar line. The gall-bladder was distended, and 3½ ounces of bile-stained fluid were withdrawn by aspiration. The common duct was greatly dilated ; the head of the pancreas was enlarged, and of stony hardness. Cholecysto-duodenostomy and choledocho-enterostomy were scarcely possible, and therefore the gall-bladder was attached to the hepatic flexure of the colon (cholecysto-colostomy) by means of the Murphy button. Three pints of saline fluid were left in the abdomen, which was then completely closed. On March 9th bile appeared in the motions, and the Murphy button was passed per rectum on March 16th. The itching disappeared, and she regained her normal colour in about a month from the time of operation. Strength was regained, and she got about actively.

Dr. Gaved Stocker informed me that she died on November 21st, 1907, with symptoms of secondary deposit in the liver, which appeared about a month previously. Until the liver mischief occurred she was quite comfortable, and there was no itching and no jaundice to the end.

**Case 3.**—Mr. P., aged 68 years, after losing flesh for some time, became rather rapidly jaundiced seven weeks before I saw him, on April 28th, 1907. The jaundice was very marked in about forty-eight hours, and had remained so since the beginning of the illness. There was no pain and no sickness. The motions were almost bile-free, and the urine deeply bile-stained. There was intense itching of the skin, causing much distress and keeping him awake at night. The loss of flesh was so rapid that he was about six pounds lighter than when weighed three weeks previously.

He looked thin and ill. The conjunctivæ were of a bright icteric tint, and the skin of a deep yellow colour. The abdomen was flat, and an elongated gall-bladder, about as large as the closed fist, could be felt to reach 2½ inches below the level of the umbilicus. The liver was enlarged to halfway between the ensiform cartilage and the umbilicus. He had suffered from piles for years, but nothing else of an abnormal character was felt per rectum. There was no enlargement of glands above the clavicle.

On May 15th an incision was made over the distended gall-bladder, which was the size of a cricket-ball, and had a " Riedel's lobe " of liver over it. No calculi were felt in the gall-bladder or ducts. The common duct was much dilated, and the head of the pancreas was firm, nodular, and as large as a small Tangerine orange. On aspirating the gall-bladder about fourteen ounces of almost black bile (sp. gr. 1012) were withdrawn. The gall-bladder was attached to the hepatic flexure of the colon by means of a Murphy button, and the abdomen completely closed after the injection of two pints of saline fluid into the peritoneal cavity.

After operation the jaundice gradually lessened and the itching of the skin entirely disappeared. The button was passed on May 30th. He gained strength, and got about actively. Later on he steadily lost flesh, and died on October 12th. Towards the end he had an occasional rigor, followed by slight jaundice, but the conjunctivæ remained clear. (Dr. Hill.)

Apart from the great comfort resulting from the absence of pruritus after such operations, I think there is some gain in the length of life, probably owing to the fact that the general toxæmia which occurs in these cases is delayed or prevented. This, however, does not admit of proof in a disease which kills so gradually, nor is it a point upon which it is necessary to lay much stress. One of my cases lived forty-six weeks after operation, and fifty-four weeks from the time of the commencement of the jaundice, and the average duration of life of the three cases is about forty-four weeks from the time of the commencement of the jaundice. This compares favourably with my experience of the duration of such cases without operation, and Fitz[1] states that " death, as a rule, rapidly follows the occurrence of jaundice and ascites. It may occur within four weeks after the former, and within six weeks after the latter."

In advising operation, the patient naturally asks what the risks are, and this is a difficult question to answer, for I do not think the cases have always been properly selected. The three cases I have quoted are all that I have operated upon for malignant disease of the pancreas, and they were all successful, but when we inquire into the statistics of a large number of cases the figures are rather disquieting. Robson and Dobson[2] quote Murphy's report, up to 1897, as showing ten deaths in twelve cases of cholecyst-enterostomy for malignant disease, which is equal to a mortality of 83.3 per cent. ; and Robson's own cases of a similar nature were seven in number with five deaths.

That the operation of cholecyst-enterostomy is not in itself responsible for this high mortality is clearly shown by the fact that Murphy performed the operation sixty-seven times, and Robson seventeen times, for non-malignant diseases without a single death. We must, therefore, look elsewhere for the reason of the marked death-rate accompanying the operation when performed for malignant disease, and it will doubtless be found associated with the existence of advanced cholæmia and toxæmia, which are known to be most prejudicial to operative success. The problem of the situation is, therefore, solved by early operation as in the three cases I have quoted above, where the average time of operation after the appearance of the jaundice was eight

1 *A System of Medicine,* edited by T. C. Allbutt, 1897, iv. 278.

2 *Diseases of the Gall-bladder and Bile-ducts.* Third Edition, 1904.

weeks. This is a most important matter, and I believe that a large measure of success can only be ensured by operating before the deposition of biliverdin in the tissues has given rise to that peculiar olive-green colour associated with the condition known as "black jaundice."[1] and which always indicates an advanced degree of cholæmia. It is desirable to decline operation in cases in which this symptom exists. In none of the three cases above recorded was "black jaundice" present, though the third case, which was operated upon ten weeks after the appearance of the jaundice, was apparently beginning to show a change of colour in the skin, which suggested its speedy occurrence if operation had not prevented it.

The measure of relief which follows cholecyst-enterostomy for malignant disease of the pancreas with jaundice and pruritus in cases in which the diagnosis is fairly positive is not, however, the only argument in favour of early operation. In spite of all that has been written on differential diagnosis by means of methods for ascertaining the presence of free glycerine in the urine, the "signe de Sahli" and other tests, it is still impossible in certain cases to distinguish between malignant disease of the pancreas and chronic interstitial pancreatitis, or even gall-stones in the common duct. Under such circumstances the patient should have the benefit of an exploratory operation, for the actual mischief may be removable by surgical measures. The difficulty of diagnosis as regards chronic interstitial pancreatitis is found even after the abdomen is opened; but in all cases of doubt we should perform cholecyst-enterostomy, which is frequently curative and associated with a very low mortality in chronic pancreatitis. It is clear, therefore, that operation is justifiable not only in certain cases of malignant disease of the pancreas when the diagnosis is positive, but also in other cases where the diagnosis is doubtful. In both circumstances the operation should be performed early, as a rule within eight weeks of the occurrence of jaundice.

The details of the operation need not be given, but there are certain points which it is desirable to mention. Reference has only been made to the attachment of the gall-bladder to the

[1] See *Encyclopædia Medica*, art. "Jaundice."

intestine (cholecyst-enterostomy), and this is the ideal operation; for though the jaundice and its accompanying symptoms would be relieved by the somewhat simpler operation of cholecystostomy, this has the disadvantages that (1) the bile is diverted from the alimentary canal, (2) there is a most troublesome biliary fistula, and (3) the mortality under similar conditions would probably be higher than in cholecyst-enterostomy. The latter procedure obviates these objections to a simple opening of the gall-bladder on the surface of the body (cholecystostomy).

Chloride of calcium is administered as a routine measure for about two days before operation, but I doubt if there is any undue tendency to hemorrhage in that stage of the disease in which operation has been recommended, though there undoubtedly is special danger from hemorrhage when the jaundice has existed for a long period.

Whether the gall-bladder should be attached to the duodenum or the colon is not of much importance. Obviously an attachment to the duodenum more closely corresponds to the natural situation for the discharge of the bile into the gut; but the comparative fixity of the hepatic flexure of the colon renders the place of union equally safe as that of the duodenum. The best plan is to take either of these two positions in accordance with the greater ease and rapidity of operation which it affords.

Rapidity of execution is one of the elements of success, and for this reason it is desirable to use a Murphy button for the union of the gall-bladder and intestine. Much as I prefer to do without any form of mechanical support in intestinal surgery generally, the Murphy button is invaluable here. Not only is there a saving of time by its use, but most operators are agreed that the mortality of cholecyst-enterostomy by direct suture is much higher than by means of the button. It is desirable to place the female part of the button in the gall-bladder, and the male part, which is heavier, in the intestine, as this arrangement favours the falling of the button into the gut when it becomes loose.

In recommending a palliative operation in so serious a condition as malignant disease of the pancreas, the wishes of the patient must be fully taken into account; but if the discomfort is great we can promise relief from jaundice and its accompanying annoyances by an operation that probably has a very low rate of mortality if performed early in the disease, and which tends to prolong life. On the other hand, if "black jaundice" has supervened, it would appear undesirable to recommend an operation which is associated with a high degree of mortality when this condition is present.

## Figures and Tables

**Figure f1:**